# The potential economic benefits of insect-based feed in Uganda

**DOI:** 10.3389/finsc.2022.968042

**Published:** 2022-11-10

**Authors:** Zewdu Abro, Ibrahim Macharia, Kelvin Mulungu, Sevgan Subramanian, Chrysantus Mbi Tanga, Menale Kassie

**Affiliations:** ^1^ International Centre of Insect Physiology and Ecology (icipe), Addis Ababa, Ethiopia; ^2^ Kenyatta University, Nairobi, Kenya; ^3^ International Centre of Insect Physiology and Ecology (icipe), Nairobi, Kenya

**Keywords:** black soldier fly, Uganda, economic impact, economic-surplus model, protein feed

## Abstract

Black soldier fly farming is gaining traction globally as a strategy for recycling organic waste into high-quality proteins and fat for feed and organic fertilizer for crop production. The support of governments in East Africa to integrate insect meal in livestock feed has opened opportunities for commercializing insect products. Understanding the potential value of Black soldier fly larvae meal (BSFLM) is paramount to inform policies and practices to promote insect farming and insect-based feed for livestock production. This paper uses the economic surplus method to generate evidence on the potential socioeconomic impact of replacing conventional soybean and fish meal protein sources with insect-based feed (IBF), BSFLM, in Uganda. Results indicate that substitution of IBF for existing protein sources will generate net economic benefits of USD 0.73 billion in 20 years (0.037 billion per year). The benefit-cost ratio is estimated at 28:1, and the internal rate of return is 138%, indicating that the insect-based animal feed industry is a profitable investment. Even in the worst-case scenario, when the replacement rate of IBF and its economic benefits are reduced by half, the benefit-cost ratio remains high (8:1). The estimated economic benefit can lift about 4.53 million people above the poverty line in the country. It can also create about 1,252─563,302 new jobs per annum, depending on the substitution rate of conventional protein feeds with IBF (0.1%─45%). Uganda has the potential to produce from about 3,244 tons to 1.5 million tons of IBF. Similarly, using the same replacement rates, the country can produce about 695─312,678 tons of NPK fertilizer from biowaste recycling. About 0.09-41 million tons of biowaste could be recycled, depending on the replacement rate of conventional feed sources with IBF. Our results justify that investing in the insect feed value chain can contribute to Uganda’s economic, social, and environmental sustainability.

## Introduction

The livestock sector in Uganda contributes about 17% of value-added in the agriculture sector and 4% of the GDP (1). The industry is the primary source of income and nutrition for 58-70% of rural households (1, 2). The cattle population in the country stands at 14 million, goats at 16 million, sheep at 5 million, poultry at 48 million, and pigs at 4 million (3). The country also produces about 100,000 tons of fish per annum (4). The annual outputs of this study’s focal animals, poultry, pigs, and fish, are valued at USD 9 million, USD 0.9 million, and USD 0.19 million, respectively (5, 6). Despite the important contributions to livelihood and the economy, the sector’s profitability is low (6).

Shortage of quality feed and high feed prices are key contributors to the country’s low profitability of poultry, pigs, and fish production (7–9). The presence of mold in feeds, adulteration, and deceptive labelling manifest in most feeds (10–12). The predominant protein sources for poultry, fish, and pig diet, which are fish meal (FM) and soybean meal (SM), are expensive, and supply will be limited in the future due to scarcity and overexploitation of resources (13). In Uganda, the price of local fish meal (cyprinid silverfish) rose from about 2,000 UGX/kg in 2005 to 5,000 UGX/kg in 2022 (14–17), while soybean meal costs about 4,500 UGX/kg (17). The overexploitation of fish from Lake Victoria has reduced fish catches by 31% (18). Soybean production is increasing in the country (6). Further, studies have demonstrated that SM has some drawbacks, including an imbalanced amino acid profile, digestive tract inflammation (19), low palatability, and anti-nutritional factors (20), limiting its inclusion level in feed formulation. Thus, dependence on FM and SM cannot be sustainable (21–23). Therefore, altrenative protein sources need to be found.

Insects that require low land and water sources have gained traction as a cheaper alternative protein source compared to FM or SM. With small footprints and environmental degradation, biofertilizers (frass) for crop production are also valuable products of insect farming (13, 21–29). Some studies have revealed that insect proteins can partially or completely replace FM and SB (30, 31).The world black soldier fly larvae market size is expected to grow by 37% per annum to reach 8,004 thousand tons (USD 3.96 billion) by 2033 (13). In Uganda and wider eastern Africa, insect meals also are predicted to grow exponentially (32, 33). It has received the backing of some national governments in East Africa, United Nations Food and Agriculture Organization (FAO), the World Bank, and the European Union (21, 28, 34, 35). Uganda has established an insect production and processing standard (36)─ with a clear policy to increase feed production and use in the livestock industry (37).

Black soldier fly larvae meal (hereafter, insect-based feed (IBF)) production is one of the fastest-growing insect value chains among all the insects available in Uganda (34). The increasing number of farmers and companies pursuing the production of IBF shows that insect farming could be a promising economic activity. The current market price of IBF (3,850 UGX/kg) is lower than the prices of FM and SM, making it an attractive feed (17). Widescale adoption of IBF farming could further reduce the current price. A great opportunity for the expansion of IBF farming exists because farmers, feed dealers, and processors have shown willingness to use IBF as feed. NGOs and research organizations are training farmers to expand insect production. Agribusiness start-ups in insect farming are emerging in the country (34). Though national production levels are unknown, some evidence suggests the presence of substantial production from the few farmers engaged in insect farming. For instance, communication with Marula Proteen Ltd, one of the biggest farms in Uganda, shows that the company produces about 80 tons of dried IBF per year.

Despite the current developments in IBF farming in Uganda, empirical evidence to justify investment in the insect protein industry does not exist. This paper fills these gaps by generating empirical evidence on the socioeconomic and environmental benefits of IBF used for poultry, pigs, and fish production. The specific objectives of the paper are to (1) undertake a gross margin analysis of insect farming based on a survey of IBF farmers; (2) estimate the potential socioeconomic and environmental benefits of substituting conventional protein sources with IBF for poultry, pigs, and fish production using the economic surplus model; and (3) assess the returns to IBF Research for Development (R4D) investment. This paper will stimulate further research and debate on how to best promote insect agriculture and insect-based feed in a way that can bring economic, social, and environmental sustainability.

## Materials and methods

### Data sources

We used data from various sources. The survey of 14 IBF farmers in Kayunga district, Central Uganda, is the first data source to compute gross margins. Most farmers are male (77%), with an average age of 29 years and education of 13 years, indicating young and educated farmers participating in the industry. Farming experience in livestock production was about eight years, and they kept an average of 56 chickens, 12 pigs, and 112 fish in one pond. Most of them started IBF farming in 2020. Country-level parameters such as eggs, chicken meat, fish, and pork production were obtained from FAOSTAT. The data on changes in yield and production costs due to IBF technology and the elasticity of supply and demand for animal products are accessed from published and non-published (e.g., expert opinion) materials (see **Table 1** below). Data on the amount of R4D investment of IBF was obtained from the International Centre of Insect Physiology and Ecology (*icipe*) and Marula Proteen Ltd. We used expert opinion to elicit the replacement rates (**Table 1**). Previous impact assessment studies use expert and local knowledge to project adoption patterns (50–54).

**Table 1:** Parameters used to calibrate equations (1), (2), and (3).

Parameters	Mean	Sources
Chicken meat yield changes due to IBF (%)	15	(38)
Eggs yield changes due to IBF (%)	1	(39–41), expert estimates
Chicken meat production cost changes due to IBF (%)	-8	(38), expert estimates
Eggs production cost changes due to IBF (%)	-23	(39–41), expert estimates
Fish production cost changes due to IBF (%)	-40	Experts and farmers estimate**^a^**
Pork production cost changes due to IBF (%)	-32	Experts and farmers estimate**^a^**
Supply elasticity for chicken meat	0.40	(42)
Supply elasticity for eggs	0.40	(42)
Supply elasticity for fish	0.80	(7)
Supply elasticity for pork	0.40	(42)
Demand elasticity for chicken meat	-0.62	(7)
Demand elasticity for eggs	-0.74	(43)
Demand elasticity for fish	-0.86	(7)
Demand elasticity for pork	-0.59	(44)
Price of chicken meat (USD/ton)	4,893	(6) and farm gate price
Price of eggs (USD/ton)	2,113	(6) and farm gate price
Price of pork (USD/ton)	4,200	(6, 45); farm gate price
Price of fish (USD/ton)	1,255	(6) and farm gate price
Chicken meat production (ton)	65,717	(6)
Egg production (ton)	44,400	(6)
Pork production (ton)	122,630	(6)
Fish production (ton)	117,590	(6, 46, 47)
Chicken Meat consumption (tonne)	65,648	(6)
Chicken meat consumption growth (%)	3.78	(48)
Egg consumption (ton)	43,893	(6)
Egg consumption growth (%)	2.60	(6, 48)
Pork consumption (ton)	122,647	(6)
Fish consumption (ton)	1,255	(6)
Discount rate (%)	12	(49)
Replacement rates (%)	0.1-45	Experts estimate**^a^**

The volume and price parameters are averages of five years (2015–2019) to smooth out shock-induced changes in product output, consumption, and prices; **^a^** the changes in production costs for fish and pork were calculated as the difference in production costs when farmers used conventional feeds and IBF; a virtual workshop was undertaken to validate these estimates by extension officers who were involved in IBF promotion.

### Valuing the economic benefits of IBF

To estimate the potential economic benefits of IBF R4D in Uganda, we use the economic surplus model (ESM). The ESM quantifies potential benefits that go to consumers and producers (50). It assumes that anytime new technologies (IBF in our case) are widely adopted, it could directly benefit producers by increasing productivity or reducing production costs and indirectly benefit consumers by lowering the prices of animal products. The benefits to consumers and producers depend on the market or trade assumption. In the absence of international trade for commodities considered in this study, the benefit of the IBF replacement is shared between producers and consumers. A closed economy assumption is plausible in the context of Uganda because the import and export of pork, eggs, chicken meat, and fish are negligible (6). In a closed economy, a technology-induced supply increase in the volume of the products would reduce the equilibrium price of livestock products and fish.

IBF technology directly impacts livestock farmers by improving productivity, lowering production costs to producers by lowering the price of protein feeds, and indirectly benefitting consumers by lowering the price of eggs, chicken meat, pork, and fish. IBF could be fed directly or by formulating it with conventional protein sources. In this study, we assume the IBF is adopted as a standalone feed because it is shown to provide the necessary energy, fat, and protein to livestock (38, 55). Emerging studies have reported that IBF’s protein and amino acid profile are comparable with fish and soybean meals (56).

We calculate the total change in economic benefits of adopting IBF in two steps. First, we calculate the K-shift parameter (50). The K-shift parameter is the proportional shift in the supply curve or the per-unit production cost decrease owing to IBF. The K-shift parameter is defined in equation (1):


(1) $$K_{m}=\left(\frac{ATT_{ym}}{\epsilon }-\frac{ATT_{cm}}{1+ATT_{ym}}\right)\times r,$$

where the index *m* stands for the livestock products: eggs, chicken meat, fish, and pork; *ATT_y_* represents the proportionate change in productivity due to IBF; *ATT_c_* is the proportionate change in the cost of production due to IBF use; *ϵ* is the elasticity of supply of livestock products; *r* is the replacement rate of IBF.

Second, we calculate the economic benefits to producers and consumers due to the use of IBF using equations (2) and (3) below.


(2) $$\text{Δ}PS_{m}=P_{m}Q_{m}\left(K_{m}-Z_{m}\right)\left(1+0.5Z_{m}\eta_{m}\right),$$

(3) $$\text{Δ}CS_{m}=P_{m}Q_{m}Z_{m}\left(1+0.5Z_{m}\eta_{m}\right),$$

where *ΔPS* and *ΔCS* indicate the benefits to producers and consumers, respectively; *P_m_* is the average producer price of product *m*; *Q_m_* represents the average production of product *m*; *Z_m_* is the relative change in prices of each product (*Z_m_* = *Km* ×*∈*/(*∈* + *η_m_*)); and *η_m_* is the absolute price elasticity of demand. The sum of *ΔPS_m_* and *ΔCS_m_* provides the change in total economic benefits due to IBF.

To estimate equations (1), (2), and (3), we use the Dynamic Research Evaluation for Management software (DREAMpy) (57). The DREAMpy is open-source software for evaluating the economic impacts of agricultural research and development using the economic surplus model. The analysis assumes a planning horizon of 20 years (2017-2036). We discounted the benefits using a 12% discount rate.

**Table 1** provides a detailed description of the parameters used to calibrate equations (1), (2), and (3). Because IBF is a new technology, the current replacement rate is limited. Based on expert estimates, Uganda’s current replacement rate of IBF is 0.1%. Furthermore, experts estimate that the replacement level of IBF will reach 45% by 2030. Using the 0.1% replacement rate of IBF in 2017 and the maximum replacement rate of 45% in 2030, replacement rates over the years were predicted using a logistic curve (50), which is inbuilt into the DREAMpy software. The underlining assumption is that substitution starts slowly and then speeds up as more farmers become aware of the benefits of the technology. Furthermore, we calculate the growth rate of the demand for chicken meat, egg, fish, and pork using data from FAOSTAT. Since the parameters are highly uncertain in ex-ante analyses and greatly influence the magnitude of the changes in the total net economic benefits, a sensitivity analysis was carried out on different parameters, including adoption rates.

### Estimate returns to IBF research

The economic surplus analysis provides information on the economic benefits of IBF R4D. However, developing the IBF products involve costs such as personnel, supplies, and demonstration, which we account for to derive the net returns. We combine the total change in economic surplus derived above with discounted research and dissemination costs to measure returns to IBF R4D using the benefit-cost ratio (BCR) and internal rate of return (IRR) indicators. When the technology’s BCR is greater than or equal to one and IRR exceeds the current interest rate, the use of IBF is deemed acceptable.

*icipe* and its partners have started IBF R4D since 2016. The total investment cost of IBF R4D was about USD 24 million between 2016 and 2019. The expenses include the investment in research, extension, training, and installing insect farming facilities. About 50% of the investment was mainly for establishing insect rearing facilities (personal communication with CEO of Marula ProTeen Limited Company). The expenses were obtained from the Finance Department of *icipe*, and Marula ProTeen Limited Company (https://weareproteen.com/) in Uganda.

### Potential poverty reduction effects of black soldier fly farming

With many funders and governments focusing on poverty reduction, tracking the effects of IBF technology on poverty reduction is a logical extension of the economic surplus method. IBF can help to mitigate poverty in a variety of ways. First, it can directly contribute to poverty reduction by increasing earnings. Second, it can reduce poverty indirectly by lowering animal product prices for consumers and increasing employment in the value chain. To estimate IBF’s potential poverty reduction effects, we use the growth elasticity of the poverty approach shown in equation (4) below.


(4) $$\Delta N\;=\left(\frac{\text{ES}}{LGDP}\times \delta \right)\times NP,$$

where ΔN is the number of people that can be lifted above the poverty line; *ES* stands for the total economic benefits of IBF farming; *LGDP* is Uganda’s livestock gross domestic product; The *LGDP* was calculated as USD 4.87 billion based on the share of the livestock GDP from the country GDP (4); *δ* is the poverty elasticity to *LGDP* (-1.58) (58); *NP* represents the number of people who live below the poverty line in Uganda (19 million) (4, 59).

### Potential employment benefits

As an emerging economic activity, IBF farming will create jobs, vital for Uganda, where youth unemployment is high. The overall unemployment rate in Uganda is 9%, while the youth unemployment rate is 13%. Following Abro et al. (60), we estimate the potential employment benefits of IBF farming using equations (5) below.


(5) $$N\;=\left(\frac{l\;\times \;r\;\times \;f}{J}\right),$$

where *N* is the number of persons who will be directly engaged in IBF production per annum; *l* is the number of labor hours needed to create one ton of IBF, estimated as 815 hours (60); IBF replacement rate of conventional feeds is denoted by *r* (%); *f* is the animal feed demand (3.2 million tons), estimated based on Abro et al., (60)’s approach; *J* is the total labor hours per year (2,496 hours). We performed the labor hour calculation considering that a worker works 26 days a month on an insect farm for 12 months at 8 hours per day.

## Results and discussion

### Gross margin analysis of insect-based feed farming

**Figure 1:**
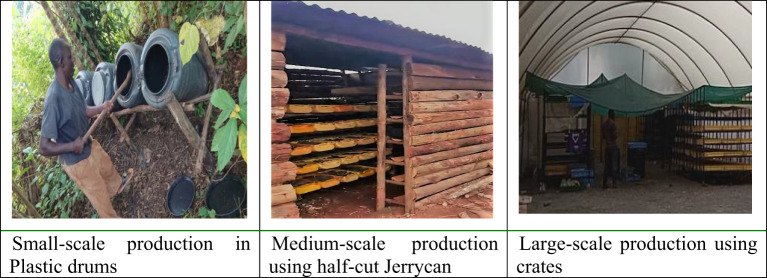
IBF production systems in Uganda (*photo taken by Abbey Lubega Akampa)*.

The survey reveals that farmers follow three production systems of IBF farming: (1) a plastic drum system used by small-scale farmers, (2) a modified greenhouse system by medium-scale farmers using half-cut jerrycans, and (3) a greenhouse system by large-scale farmers (**Figure 1**). All the farmers reported that they bought the first instar larvae. Farmers also said that they produce IBF in eight production cycles. The three primary substrates used by small and medium-scale farmers are food and farm wastes mixed with pig and chicken waste. The large-scale IBF farmers use wastes from markets and the fruit juice industry, and most farmers practice integrated farming: IBF, chicken, crops, and pig production. Only two of the farmers reported support from the government extension. Farmers mainly feed the live larvae for their livestock. Only two IBF farmers reported sun-dry the larvae before feeding their livestock. Considering eight production cycles per year, the total production of IBF by the surveyed farmers was about 232,464 kg live larvae, with an average production of 4,291 kg for small-scale farmers, 25,160 kg for medium-scale farmers, and 26,973 kg for large-scale farmers.

**Table 2** shows the three production systems’ gross margins and costs associated with IBF farming. The key costs of the IBF farmers were labor, substrates, and purchase of first instar larvae. Regardless of the production system, the gross margins are positive. However, small-scale farmers obtain the highest GM ratio (65%) compared to medium-scale (55%) and large-scale (52%) farmers. The gross margin analysis shows that IBF farming seems profitable. Using this estimate as an indication, we present the potential country-wide benefits of scaling IBF farming in Uganda in the following subsections.

**Table 2:** Average gross margin analysis for IBF farming in Uganda.

Production scale			
Variables	Small-scale	Medium-scale	Large-scale
Revenue (USD/cycle)	48	485	312
	(10)	(107)	(76)
Sale of frass (USD/cycle)	8	45	589
	(2)	(12)	(89)
IBF produced (tons)	4.30	25.16	26.97
	(1.32)	(8.32)	(3.07)
Production Cost (USD/cycle)	16	214	277
	(2)	(27)	(14)
Buying of first instar larvae (USD/cycle)	5	14	19
	(0.00)**^¥^**	(0.00)**^¥^**	(0.00)**^¥^**
Feeding and other costs (USD/cycle)	4	178	236
	(0.41)	(26)	(13)
Labor (USD/cycle)	7	22	22
	(1.35)	(1.03)	(1.18)
Gross Margin (USD/cycle)	32	270	312
	(10)	(100)	(76)
GM ratio (%)	65	55	52
	(7.79)	(7.84)	(5.33)

Standard deviations are in brackets; The average exchange rate during the survey was 3,681 UGX/USD; **^¥^** the standard deviations are zero because the price is the same in each group.

### Economic benefits to producers and consumers

**Table 3** presents the net economic benefits of IBF (total change in economic surplus minus IBF research and demonstration costs (USD 24 million)) for 20 years (2017-2036). We use a research lag of 5 years (2017-2021) to predict the economic impact of the replacement rate. The total economic benefits from replacing conventional protein sources with IBF are estimated at USD 0.73 billion (USD 0.037 billion per year). The most significant portion of these benefits goes to producers (59%). The total benefit is about 28 times more than the amount spent on IBF research and extension. Findings illustrate that investing in IBF research is profitable, with internal rates of return of 138% and a benefit-cost ratio of 28:1. Benefits differ substantially across livestock products, with a more significant proportion of the benefits accruing to pig production and the lowest to egg production.

**Table 3:** Expected economic surplus of substituting conventional protein feeds with IBF (2017-2036).

LivestockProduct	Discounted net economic benefit (billion USD)			Costs discounted (billion USD)	Benefit/Cost ratio		Internal rate of return (%)
	Producer	Consumer	Total			
Chicken	0.18	0.12	0.30	0.01	39	145	
Eggs	0.03	0.02	0.05	0.00	15	138	
Fish	0.06	0.06	0.12	0.00	25	111	
Pigs	0.16	0.10	0.26	0.01	32	156	
Total (average)	0.43	0.30	0.73	0.02	28	138	

### Poverty reduction effects

Based on equation (4), the estimated potential poverty reduction effects of IBF farming are reported in **Table 4**. We find that the estimated economic benefits of IBF can potentially reduce the number of people below the poverty line by 4.53 million in the study’s 20 years. The reduction is 0.23 million per annum, which is equivalent to 1.19% of the annual total number of people who live below the poverty line.

**Table 4:** Impact of substituting conventional protein sources by IBF on poverty reduction.

Variables	Estimated values
Total benefits from IBF in Uganda (Billions of USD)	73
Livestock GDP in Uganda (Billions of USD)	486
Total population in Uganda (millions)	46
Poor people (%) in Uganda	0.42
Poor people (number) in Uganda (millions)	19
Elasticity of poverty in Uganda	1.58
Number of people lifted above the poverty line (20 years)	4.53
Number of people lifted above the poverty line per annum	0.23

### Employment benefits

**Figure 2:**
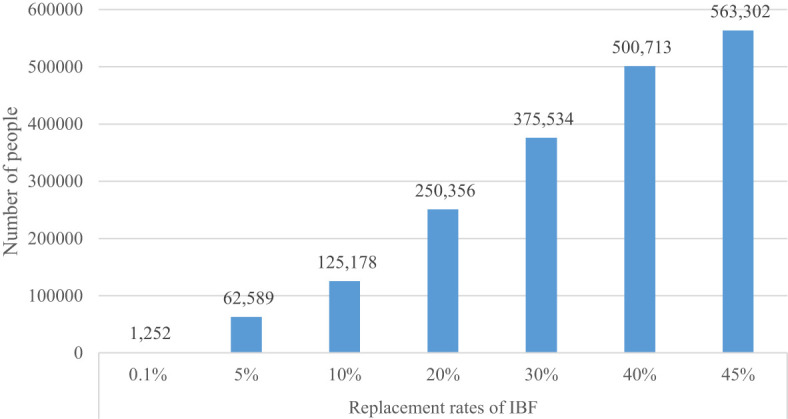
Number of jobs created.

Using equation (5), we have estimated the potential employment effects of substituting conventional sources by IBF. Since the replacement rate of IBF is important to determine the number of people that can be potentially employed, we used various replacement rates. At the replacement rate of 0.1%, the number of jobs that could be created is about 1,252 (**Figure 2**). As the replacement rate increases, the number of people employed increases significantly. For instance, IBF could generate new jobs for 62,589 people at a 5% replacement rate and 563,302 people at a replacement rate of 45%. Given the high unemployment rate among the youth, introducing IBF can provide enormous prospects for new job creation. This estimate could be a lower bound as it reflects direct effects only. Due to data limitations, we did not account for potential employment along the value chain, such as marketing of BSFL, processing and marketing of bio-fertilizers, and activities of input suppliers.

### Estimate IBF and biofertilizer production

**Figure 3:**
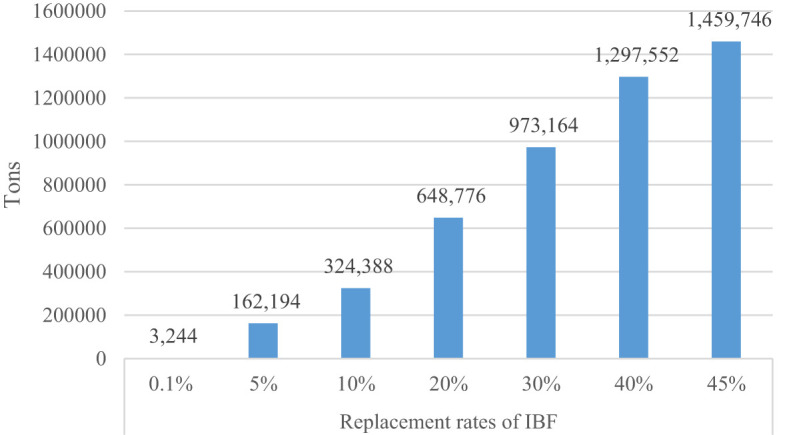
Total IBF production.

The important primary product of black soldier fly farming is protein meal production. The volume of protein meal production depends on its substitution rates for the existing feed sources. Uganda’s total demand for feed for poultry, pig, and fish production is about 3.2 million tons per annum, estimated based on Abro et al. (60)’s approach. To fulfil this demand, we use various replacement rates of IBF, ranging from a minimum of 0.1% to a maximum of 45%. Therefore, the total IBF that could be potential produced is obtained by multiplying the replace rates by total feed demand and dividing it by 100. **Figure 3** shows the total IBF production at various substitution rates. Uganda can produce 3,244 tons of IBF at a 5% substitution rate of conventional feed. At a 45% demand for IBF, the country can produce 1.5 million tons of dried IBF.

**Figure 4:**
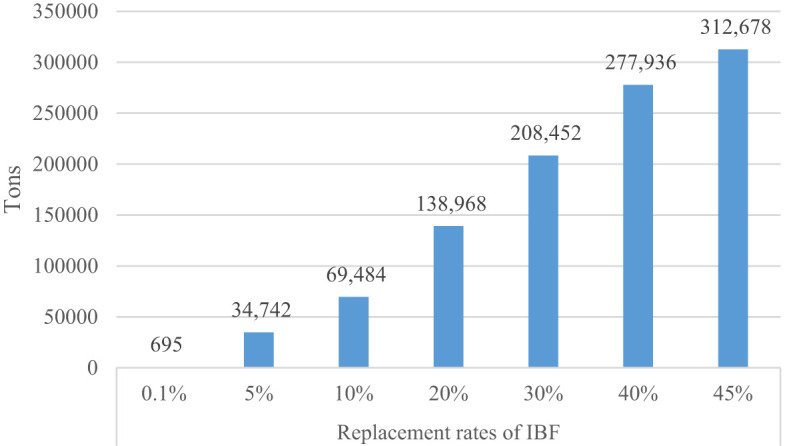
Biofertilizer (NPK equivalent) production.

Next to IBF, biofertilizer is a key by-product of IBF farming. To understand the potential of IBF farming on biofertilizer production, we estimated the NPK equivalent fertilizers that could be produced from waste recycling. According to Abro et al. (60), for each ton of IBF produced, 6 tons of frass could be generated. And each ton of frass carries 2.14% Nitrogen, 0.85% Phosphorus, and 0.58% potassium. Based on these parameters, the total NPK fertilizer that can be potentially produced is reported in **Figure 4**. At the replacement rate of 0.1%, Uganda has the potential to produce about 695 tons of NPK. As the replacement rate increases, the NPK fertilizers that could be produced dramatically increases. Local production of biofertilizers is paramount to strengthen the local food systems in the face of foreign currency constraints and external shocks, such as the Russia-Ukraine war that disrupts fertilizer supply and price.

### Waste management effects

**Figure 5:**
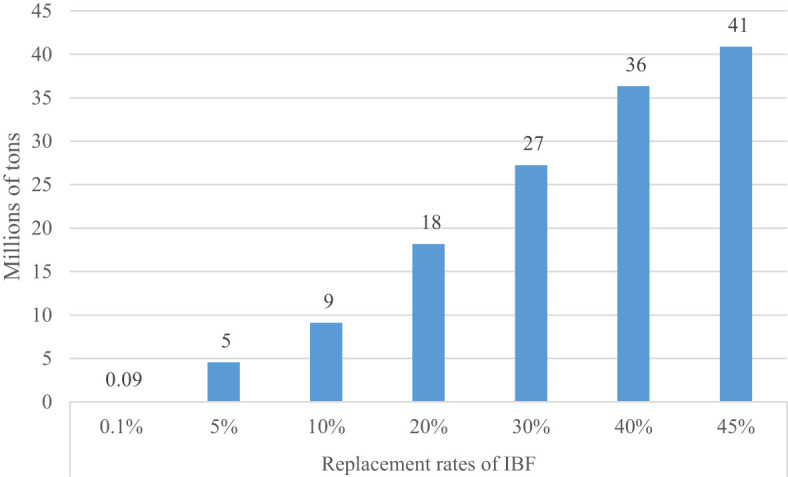
Biowaste that could be recycled using insect farming.

Uganda generates about 1.4 million tons per annum of agro-processing waste and bagasse (61). It also generates 6.4 million tons of farm-level agricultural leftovers (62). Aside from mango and other vegetal wastes, it is estimated that coffee hullers in Uganda create 0.28 million tons of coffee husks per annum (63). Currently, the total estimated household biowaste in Uganda is about 5-7 million tons per year (64, 65). Most of these biowastes are not recycled. These wastes are disposed of in landfills or burned, which has detrimental effects on health and the environment. This type of contamination would be greatly reduced by IBF farming. To produce 1 ton of dried IBF, it will need 28 tons of wet biowaste (60). Using this conversion rate, we estimated the total biowaste that could be recycled at different replacement rates of IBF. Substituting 0.1-45% of the conventional protein feeds with IBF feed would need recycling 0.09-41 million tons (**Figure 5**), a significant environmental clean-up service if the government and private sector actors promote IBF farming.

### Sensitivity analysis

We conducted a sensitivity analysis to demonstrate the robustness of the estimated benefits. We change the key parameters of interest that drive the K-shift parameter holding other parameters constant. The key parameters changed were the yield benefit, cost reduction, and the replacement rate of IBF. This can also be used to set the thresholds of the parameters below which the benefits will disappear. This is conducted under two main scenarios: a) reducing the baseline value by 50%, and b) a worst-case scenario where all the parameters are changed simultaneously, assuming a low replacement rate and poor performance of IBF.

Results reveal that the estimated net economic benefit is about USD 0.21 billion, with a BCR of 18:1, considering the first scenario (**Table 5**). This implies that even when the IBF benefits reduced by half, the technology still generates substantial economic benefits. Increasing the production cost by 50% of the base value reduces the economic benefits by 33% to USD 0.49 billion. Further, reducing the replacement rate by 50% results in USD 0.38 billion. Decreasing yield by 50% and simultaneously increasing the production cost generates USD 0.35 billion while increasing the cost of production by half and a 50% reduction in replacement rate gives USD 0.29 billion. Finally, under an improbable scenario (the worst-case scenario) with all variables reduced by half, the total economic benefit is estimated at USD 0.21 billion with a BCR of 8:1.

**Table 5:** Sensitivity analysis of the economic impact of IBF in Uganda (2017-2036).

Scenarios	Description	Product	Net economic benefit (billion USD)			Costs discounted (billion USD)	Benefit-cost ratio
			Producer	Consumer	Total		
1	50% reduction in yield advantage	Chicken	0.10	0.06	0.16	0.01	20.78
		Eggs	0.03	0.02	0.05	0.00	14.65
		Total	0.13	0.08	0.21	0.01	17.72
2	50% increase in production cost	Chicken	0.16	0.10	0.27	0.01	34.88
		Eggs	0.02	0.01	0.03	0.00	8.70
		Fish	0.04	0.03	0.07	0.00	14.28
		Pig	0.08	0.05	0.13	0.01	15.77
		Total	0.29	0.20	0.49	0.02	18.41
3	50% reduction in the replacement rate	Chicken	0.09	0.06	0.15	0.01	19.86
		Eggs	0.02	0.01	0.02	0.00	7.81
		Fish	0.04	0.03	0.07	0.00	14.60
		Pig	0.08	0.05	0.13	0.01	16.13
		Total	0.22	0.15	0.38	0.02	14.60
4	50% reduction in yield and a 50% increase in production cost	Chicken	0.08	0.05	0.13	0.01	17.28
		Eggs	0.02	0.01	0.02	0.00	7.97
		Fish	0.04	0.03	0.07	0.00	14.28
		Pig	0.08	0.05	0.13	0.01	15.77
		Total	0.21	0.15	0.35	0.02	13.83
5	50% increase in production cost and a 50% reduction in the replacement rate	Chicken	0.11	0.07	0.18	0.01	12.80
		Eggs	0.01	0.00	0.01	0.00	4.43
		Fish	0.02	0.02	0.03	0.00	7.25
		Pig	0.04	0.03	0.07	0.01	8.03
		Total	0.18	0.12	0.29	0.01	8.13
6	Reducing yield and replacement rate by 50%, and 50% increase in production cost	Chicken	0.06	0.04	0.10	0.01	12.80
		Eggs	0.01	0.00	0.01	0.00	4.06
		Fish	0.02	0.02	0.03	0.00	7.25
		Pig	0.04	0.03	0.07	0.01	8.03
		Total	0.13	0.09	0.21	0.01	8.04

We also analyzed the sensitivity of the potential poverty reduction effects of IBF farming. The formula used to estimate the potential poverty reduction effects depends on the country’s total number of poor people, the economic benefit generated from IBF, the growth-poverty elasticity parameter, and the number of poor people in Uganda. The key challenge is that we cannot directly estimate the growth-poverty elasticity estimate due to data limitations. For this reason, we used the elasticity of poverty concerning growth in agricultural GDP, the closest and most approximate estimate in the literature. Previous literature uses the elasticity of poverty to agricultural GDP to estimate the poverty of impacts of development interventions (53, 60, 66) To understand the sensitivity of the number of people that could be lifted above the poverty line, we varied the poverty elasticity parameter in 3 scenarios. These scenarios are based on previous estimates for Uganda. We use the minimum (0.06), average (1.45), and maximum (3.78) growth-poverty elasticity (67). The results are shown in **Table 6**. Even at a lower responsiveness level (0.06), the impact on poverty is still remarkable (a reduction of 0.05-0.17 million, depending on the scenarios). If poverty is highly responsive to income from IBF farming (at 3.78), the number of people that could be lifted above the poverty line is 3.12-10.83 million.

**Table 6:** Sensitivity analysis for the economic impact of IBF farming on poverty (2017-2036) in Uganda.

Sources of the economic benefit estimates	Variables	Growth poverty elasticity scenarios		
		Scenario 1 (0.06)	Scenario 2 (1.45)	Scenario 3 (3.78)
Main result-**Table 3**	Total benefit in Uganda (Billions of USD)	0.73	0.73	0.73
	Livestock GDP in Uganda (Billions of USD)	486	486	486
	Total population in Uganda (millions)	46	46	46
	Poor people (%) in Uganda	0.42	0.42	0.42
	Poor people (number) in Uganda (millions)	19	19	19
	Elasticity of poverty in Uganda	0.06	1.45	3.78
	Number of people lifted above the poverty line	0.17	4.14	10.83
**Table 5**-Scenario 1	Total benefit in Uganda (Billions of USD)	0.21	0.21	0.21
	Livestock GDP in Uganda (Billions of USD)	486	486	486
	Total population in Uganda (millions)	46	46	46
	Poor people (%) in Uganda	0.42	0.42	0.42
	Poor people (number) in Uganda (millions)	19	19	19
	Elasticity of poverty in Uganda	0.06	0.36	3.78
	Number of people lifted above the poverty line	0.05	0.30	3.12
**Table 5**-Scenario 2	Total benefit in Uganda (Billions of USD)	0.49	0.49	0.49
	Livestock GDP in Uganda (Billions of USD)	486	486	486
	Total population in Uganda (millions)	46	46	46
	Poor people (%) in Uganda	0.42	0.42	0.42
	Poor people (number) in Uganda (millions)	19	19	19
	Elasticity of poverty in Uganda	0.06	0.36	3.78
	Number of people lifted above the poverty line	0.12	0.70	7.27
**Table 5**-Scenario 3	Total benefit in Uganda (Billions of USD)	0.38	0.38	0.38
	Livestock GDP in Uganda (Billions of USD)	486	486	486
	Total population in Uganda (millions)	46	46	46
	Poor people (%) in Uganda	0.42	0.42	0.42
	Poor people (number) in Uganda (millions)	19	19	19
	Elasticity of poverty in Uganda	0.06	0.36	3.78
	Number of people lifted above the poverty line	0.09	0.54	5.64
**Table 5**-Scenario 4	Total benefit in Uganda (Billions of USD)	0.35	0.35	0.35
	Livestock GDP in Uganda (Billions of USD)	486	486	486
	Total population in Uganda (millions)	46	46	46
	Poor people (%) in Uganda	0.42	0.42	0.42
	Poor people (number) in Uganda (millions)	19	19	19
	Elasticity of poverty in Uganda	0.06	0.36	3.78
	Number of people lifted above the poverty line	0.08	0.50	5.19
**Table 5**-Scenario 5	Total benefit in Uganda (Billions of USD)	0.29	0.29	0.29
	Livestock GDP in Uganda (Billions of USD)	486	486	486
	Total population in Uganda (millions)	46	46	46
	Poor people (%) in Uganda	0.42	0.42	0.42
	Poor people (number) in Uganda (millions)	19	19	19
	Elasticity of poverty in Uganda	0.06	0.36	3.78
	Number of people lifted above the poverty line	0.07	0.41	4.30
**Table 5**-Scenario 6	Total benefit in Uganda (Billions of USD)	0.21	0.21	0.21
	Livestock GDP in Uganda (Billions of USD)	486	486	486
	Total population in Uganda (millions)	46	46	46
	Poor people (%) in Uganda	0.42	0.42	0.42
	Poor people (number) in Uganda (millions)	19	19	19
	Elasticity of poverty in Uganda	0.06	0.36	3.78
	Number of people lifted above the poverty line	0.05	0.30	3.12

## Conclusions and discussion

This study evaluates the potential socioeconomic benefits of substituting conventional dietary protein sources with insect-based feed (IBF) for poultry, pigs, and fish production. To our knowledge, we offer some of the first empirical evidence on the benefits of insect farming and IBF. This paper extends the existing evidence of IBF (e.g., 34, 60). Unlike Abro et al. (60), this paper estimates the net returns and profitability of investment in IBF research and dissemination. The paper considers pigs, poultry, and fish, whereas Abro et al. mainly focuses on poultry production. Although the sample size is small, this paper generates farm-level evidence of insect farming using gross margin analysis employing data from 14 IBF producers. Verner et al. (34) also quantified the IBF production and associated employment and environmental effects of IBF production in 11 African countries (Uganda not included). However, these authors did not quantify the economic and poverty reduction benefits of the substitution of IBF for dietary fish meal and soybean meal. Unlike Verner et al. (34), we linked production and cost changes in chicken meat, eggs, pigs, and fish production due to the introduction of IBF. Our approach enabled us to quantify the potential economic gains and aggregate welfare implications at the country level.

From this study, three important findings emerge. First, investment in insect farming has a great potential to produce a huge amount of protein feed and biofertilizers for crop production. At the lowest replacement rate of IBF (0.1%), Uganda requires to produce 3,244 tons of IBF per annum. If IBF is scaled to replace 45% of the existing protein sources, the country can produce about 1.5 million tons of IBF. Uganda has the potential to produce about 695 tons of biofertilizers at a replacement rate of 0.1% and 312,678 tons at a 45% replacement rate. The production of this biofertilizers needs recycling 0.09-41 million tons of biowaste. The production of biofertilizers could help strengthen the local food systems by mitigating the high and increasing price of inorganic fertilizers (68). Furthermore, it can fulfil the growing demand for inorganic fertilizers that have grown by 7% per annum over the last decade in Uganda (6) and divert the foreign currency being used to import fertilizer into other development activities.

Second, the net economic benefits of IBF are USD 0.73 billion for 20 years (USD 0.037 Billion per annum). This estimate is within the estimated range economic benefits in Kenya by Abro et al. (60), who reported USD 16-159 million for the commercial poultry sector in Kenya. With insect-based feed project costs of USD 24 million, the benefit-cost ratio was estimated at 28:1 and an internal rate of return of 138%, indicating that investment in the insect industry will be highly profitable.

Third, the economic benefits of promoting IBF have the potential to address multiple sustainable development goals simultaneously (e.g., no poverty, zero hunger, decent work, economic growth, gender equality, and climate action). The economic benefits of USD 0.73 billion can lift about 4.53 million people (0.23 million per annum) above the national poverty line. The reduction in the number of people below the poverty line is about 1.19% of the total number of poor people per annum. The substitution of IBF for dietary FM or SM can create employment for as low as 1,252 people at a 0.1% substitution rate and as high as 563,302 people at a replacement rate of 45%. Although results are not directly comparable, these results are qualitatively similar to Abro et al. (60), and Verner et al. (34) in that insect farming has positive socioeconomic impacts while enhancing environmental sustainability. For example, Verner et al. (34) reported that black soldier fly farming could generate employment for about 1.4-15.5 million jobs in Africa.

The key lesson from this study is that investing in IBF production can contribute to economic, social, and environmental sustainability. We have demonstrated the sensitivity of our results to changes in the assumptions. Even in the worst-case scenarios where replacement rates, costs, yield levels, and growth-poverty elasticity parameters were reduced by half, the socioeconomic potential of IBF is remarkable.

Although our estimates demonstrate the positive benefits of IBF, the study has some limitations. First, our analysis depends on data from various sources, including expert estimates. Future studies need to validate the expert estimates by collecting actual detailed data from IBF adopters in Uganda and the wider eastern Africa region, where insect farming is becoming an emerging economic activity. Besides, the cost structure used to set the price of IBF, and farm-level investment worthiness of IBF farming should be undertaken to understand how attractive IBF is to individual farmers. Second, we did not capture the potential crop yield impact of biofertilizers, an essential by-product of IBF farming due to data limitations. Such changes should be captured to understand the full impact of IBF. Third, our estimate did not capture the potential value of organic wastes used to produce feed and biofertilizers. Fourth, we used a partial equilibrium approach, which does not capture general equilibrium effects due to the expansion of IBF that may change relative feed and consumer products (e.g., eggs) prices and labor wage. Developing integrated and dynamic decision-making modeling tools is appealing to estimate both the direct and indirect effects of IBF technology after accounting for the influence of income and population dynamics on demand for the IBF.

One important policy challenge that needs to be addressed is how to expand insect farming. The transition from conventional feed sources to IBF protein may not happen immediately because developing its value chain and business model will require resources. Fear of potential risk (e.g., disease), lack of skills, and supply-side constraints such as capital that enable to process of significant waste into protein and fertilizer could be key replacement constraints. Addressing these challenges requires continuous awareness creation along the value chain to create demand, capacity building, engaging private sectors, facilitating finance, product development and marketing, policy dialogue among stakeholders, and implementing the right policy incentives. Currently, donors and research organizations are eying the potential of IBF farming for large-scale specialized farms. There is also a strong belief that IBF farming could contribute to job creation, food security, and poverty reduction for small-scale farmers by integrating it with poultry, pig, and/or fish and crop production (32, 34, 69). We recommend future studies to examine the efficiency of the small, medium, and large-scale insect farmers to achieve the promises of insect farming: low environmental footprint, higher income, generating jobs, and quality feed protein, among others.

## Data availability statement

The original contributions presented in the study are included in the article/supplementary material. Further inquiries can be directed to the corresponding author.

## Author contributions

ZA, IM, KM, SS, CT, and MK contributed to the conception and design of the study. IM organized the database and performed the statistical analysis. ZA and IM wrote the first draft of the manuscript. KM, SS, and CT review the manuscript. All authors contributed to the article and approved the submitted version.

## Acknowledgments

We gratefully acknowledge the financial support from the Australian Centre for International Agricultural Research (ACIAR) (ProteinAfrica–Grant No: LS/2020/154), the Rockefeller Foundation (WAVE-IN—Grant No.: 2021 FOD 030), Norwegian Agency for Development Cooperation, the Section for research, innovation, and higher education grant number (Grant No.: RAF–3058 KEN–18/0005) (CAP–Africa), the Curt Bergfors Foundation Food Planet Prize Award, the Swedish International Development Cooperation Agency (Sida); the Swiss Agency for Development and Cooperation (SDC); the Federal Democratic Republic of Ethiopia; and the Government of the Republic of Kenya. The funders had no role in the study design, data collection and analysis, publication decision, or manuscript preparation. We also thank the enumerators and supervisors for their dedication to conducting the surveys and the farmers and experts who participated in the study.

## Conflict of interest

The authors declare that the research was conducted in the absence of any commercial or financial relationships that could be construed as a potential conflict of interest.

## Publisher’s note

All claims expressed in this article are solely those of the authors and do not necessarily represent those of their affiliated organizations, or those of the publisher, the editors and the reviewers. Any product that may be evaluated in this article, or claim that may be made by its manufacturer, is not guaranteed or endorsed by the publisher.

## Author disclaimer

The views expressed herein do not necessarily reflect the official opinion of the donors.
